# Rapid Whole Genome Sequencing Diagnoses and Guides Treatment in Critically Ill Children in Belgium in Less than 40 Hours

**DOI:** 10.3390/ijms24044003

**Published:** 2023-02-16

**Authors:** Aimé Lumaka, Corinne Fasquelle, Francois-Guillaume Debray, Serpil Alkan, Adeline Jacquinet, Julie Harvengt, François Boemer, André Mulder, Sandrine Vaessen, Renaud Viellevoye, Leonor Palmeira, Benoit Charloteaux, Anne Brysse, Saskia Bulk, Vincent Rigo, Vincent Bours

**Affiliations:** 1 Human Genetic Laboratory, GIGA Institute, University of Liège, 4000 Liège, Belgium; 2 Center for Human Genetics, Centre Hospitalier Universitaire, 4032 Liège, Belgium; 3 Neuropediatric Division, CHU de Liège—CHR de la Citadelle, University of Liège, 4000 Liège, Belgium; 4 Department of Pediatrics, Division of Pediatric Critical Care Medicine, CHC Mont-Légia, 4000 Liège, Belgium; 5 Neonatology Division, CHU de Liège—CHR de la Citadelle, University of Liège, 4000 Liège, Belgium

**Keywords:** rapid whole genome sequencing, rWGS, critically ill newborn, NICU, NICU-Seq, Dragen, Base Space Sequence Hub

## Abstract

Rapid Whole Genome Sequencing (rWGS) represents a valuable exploration in critically ill pediatric patients. Early diagnosis allows care to be adjusted. We evaluated the feasibility, turnaround time (TAT), yield, and utility of rWGS in Belgium. Twenty-one unrelated critically ill patients were recruited from the neonatal intensive care units, the pediatric intensive care unit, and the neuropediatric unit, and offered rWGS as a first tier test. Libraries were prepared in the laboratory of human genetics of the University of Liège using Illumina DNA PCR-free protocol. Sequencing was performed on a NovaSeq 6000 in trio for 19 and in duo for two probands. The TAT was calculated from the sample reception to the validation of results. Clinical utility data were provided by treating physicians. A definite diagnosis was reached in twelve (57.5%) patients in 39.80 h on average (range: 37.05–43.7). An unsuspected diagnosis was identified in seven patients. rWGS guided care adjustments in diagnosed patients, including a gene therapy, an off-label drug trial and two condition-specific treatments. We successfully implemented the fastest rWGS platform in Europe and obtained one of the highest rWGS yields. This study establishes the path for a nationwide semi-centered rWGS network in Belgium.

## 1. Introduction

Genetic diseases are recognized as an important cause of admission and death in neonatal and pediatric intensive care units [1,2], and the proportion of genetic diagnoses is the highest among critically ill patients [3]. Early testing helps in diagnosing those diseases, and providing useful information at a time when it is still possible to modify the path of the disease. The traditional hypothesis-driven diagnostic approach may not be efficient in these patients, since the clinical expression during the neonatal period is often nonspecific, and phenotypes with reduced survival for many genetic diseases are not fully clinically recognizable because of very early deaths [4]. In addition, the standard turnaround time (TAT) for genome-wide sequencing tests (Mendeliome or exomes) does not allow the diagnosis to impact most clinical decisions in a timely fashion. Therefore, severely ill neonatal and infant populations may be considered an uncharacterized niche for early-onset severe and lethal forms of known diseases and for early onset of yet-unknown lethal diseases, for which hypothesis-free genetic testing would be required.

Whole Genome Sequencing (WGS) has been widely proven to be the best exploration method in critically ill pediatric patients, as it allows a hypothesis-free process and the exploration of multiple types of genetic defects, including single nucleotide variants (SNVs), copy number variations (CNVs), complex structural variants (SNVs), repeat expansions and mosaic aneuploidies [3,5,6,7,8,9]. A few teams worldwide are progressively compressing the time from sampling to the return of results (turnaround time, or TAT) of the WGS for critically ill pediatric patients. The current TAT of this fast-track WGS, also referred to as rapid or ultra-rapid whole genome sequencing (rWGS), varies from barely more than 7 h to a few weeks, depending on the workflow and the sequencing technology [3,5,10,11,12,13]. Although these improvements have mostly been reported by USA teams, reports on the implementation of rWGS in understudied populations are also surfacing [5]. The shortest reported TATs in Europe thus far, regardless of the approach (exome sequencing or genome sequencing), ranged between 5 and 21 days depending on the study [5,14,15,16].

According to recent statistics, about 113,000 births were recorded in Belgium in 2020 [17], and about 10% of those newborns were admitted to neonatology units, including about 3.8 to 5.2% in Neonatal Intensive Care Units (NICU) [18]. Based on those statistics, a non-negligible fraction of newborns in Belgium is potentially eligible for fast-track genomic testing for diagnosis and care. We, therefore, initiated the present pilot study aiming to evaluate the feasibility, time to diagnosis, yield, and utility of rWGS in Belgium.

## 2. Results

### 2.1. Demographic and Enrollment Criteria

Twenty-one (11 girls and 10 boys) unrelated patients were recruited and offered rWGS. The median age at recruitment was 0.27 years (98.62 days, range, 2 days–18 years), nine patients (42.86%) were admitted to NICU, six (28.57%) to Pediatric Intensive Care Unit (PICU), and the remaining six (28.57%) to neuropediatric ward (NEURO) (Table 1). None of them received a postnatal genetic test before their recruitment. Given NIPT has been offered systematically in Belgium since April 2017, we assumed that the NIPT was performed in the 18 patients born in Belgium and was normal. We did not find a record of invasive of prenatal testing in any of the patients.

Our inclusion check list (Table S1), slightly adapted from the Blue Shield checklist, comprised nine major clinical categories: (1) developmental delay/mental retardation; (2) specific anomaly highly suggestive of a genetic etiology; (3) an abnormal laboratory test suggests a genetic disease or a complex metabolic phenotype; (4) an abnormal response to standard treatment for a major underlying condition; (5) significant hypotonia; (6) persistent seizures; (7) infant with high-risk stratification on assessment of a Brief Resolved Unexplained Event (BRUE); (8) significantly abnormal electrocardiogram (ECG); and (9) positive family history of the above 8 criteria. Sixteen different congenital malformations were encountered among these 21 patients (Table S2). The single most common indication for rWGS was severe hypotonia (10 patients, 47.72%), whereas abnormal biochemical status and persistent seizures followed, with 38.09 (eight patients) and 23.81% (five patients), respectively.

### 2.2. Molecular Diagnostic Yield

**Figure 1:**
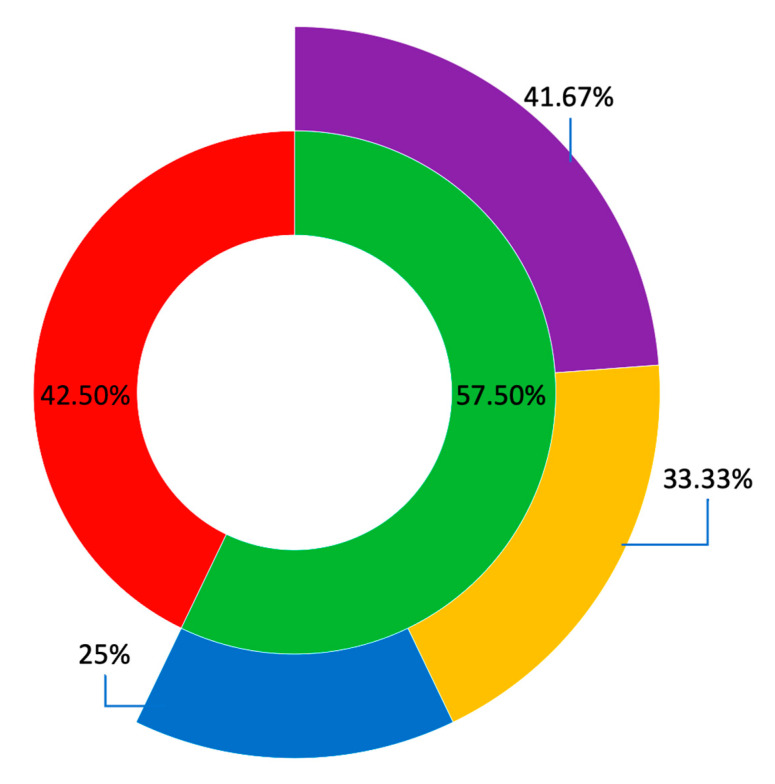
Diagnostic yield of rWGS in this cohort. Legend: the inner donut presents the solved cases (green) and the unsolved cases (red). The outer donut spreads the solved cases across the three recruitment units, namely NICU in purple, PICU in orange and NEURO in blue.

Molecular diagnoses were reached for 12 probands (57.50%), including five probands from NICU (41.67% of solved cases), four from PICU (33.33%), and three from the NEURO (25.00%) (Figure 1). The yield per unit was 5/9 (55.56%) for patients enrolled in NICU, 4/6 (66.67%) in PICU, and 3/6 (50%) in the NEURO.

In all 12 solved patients, the molecular defect was a SNV. The analyses of CNVs and mitochondrial DNA captured with the nuclear DNA during the library preparation, did not return additional diagnoses (Table 2).

Interestingly, one patient (rWGS_6) presented with two different molecular diagnoses (1/21, 4.76%), each explaining part of the phenotype. In another patient (rWGS_5), we identified 3 variants in the *PIEZO* gene, among which one was inherited from the mother, one from the father and one de novo. Further analyses are planned to determine on which allele the de novo variant occurred.

The molecular diagnosis validated the clinical hypothesis in five patients: hereditary Lymphedema (rWGS_5), mitochondrial depletion syndrome (rWGS_9), Alagille syndrome (rWGS_14), *TUBB*-related spectrum syndrome (rWGS_18) and 3-hydroxy-3-methylglutaryl-Coa lyase deficiency (rWGS_19).

### 2.3. Turnaround Time

**Figure 2:**
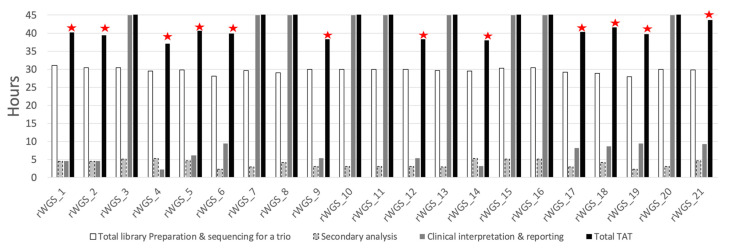
Time metrics of rWGS in the 21 patients. Legend: for each patient, the white bar indicates library preparation, sequencing, and demultiplexing time, the grey bar with dashed borders represents the secondary analysis (from fastq to vcf), whereas the darker grey bar corresponds to the clinical interpretation time. Though the scale of the graph is limited to 45 h, the analysis was closed after a total of 7 days in unsolved patients. The red stars indicate patients molecularly diagnosed in this study.

The average time from the sample reception in the laboratory to the signature of the report was 39.80 h (ranges, 37.05–43.7) in solved patients (Figure 2). The shortest TAT in this series was 37.05 h (rWGS_4). In unsolved patients, the clinical interpretation was deliberately kept open for one week in total to include 5 days of additional clinical investigation of extra abnormalities such as repeat expansion and Run Of Homozygosity (ROH). In all patients, the report was returned prior to hospital discharge.

The longest component varied depending on the resolution status. For solved patients, the sequencing run time for a S1 PE300 cycles on the NovaSeq 6000 (25 h) was the longest component. In unsolved patients, the further clinical analysis and interpretation time of about 5 days constituted the longest component. Of note, none of the prolonged analysis led to a conclusive diagnosis.

### 2.4. Clinical Utility

Based on the rWGS results, an adjustment was made to the clinical management for the 12 diagnosed patients, consisting of inclusion in a clinical trial for two, implementation of a disease-specific treatment for two, and adjustment of multidisciplinary care in the remaining eight patients.

Of the two patients included in clinical trials, one patient (rWGS_2) was diagnosed with Aromatic l-amino acid decarboxylase deficiency (AADCD, MIM #608643). Their treatment first consisted of Pramipexole, selegiline, and Artane with a non-satisfactory response. At an age of 30 months, he was included in an in vivo gene therapy trial using an adeno-associated virus 2 delivery of human aromatic L-amino acid decarboxylase (AAV2-hAADC) in the nervous system (Upstaza, 2.8 × 1011 genome vector, from PTC Therapeutics^TM^). The evaluation performed six months after treatment indicated significant clinical improvement. The second patient to be included in a clinical trial (rWGS_17) was diagnosed with developmental and epileptic encephalopathy type 14 due to de novo mutation in *KCNT1* (MIM #614959). Treating clinicians initiated an off-label clinical trial with quinidine. Dose escalation is still going on at the time of the submission of this manuscript.

The disease-specific treatment consisted of Leucine restricted diet in a patient (rWGS_19) with HMG-CoA lyase deficiency (MIM #246450) and pancrelipase in a homozygous proband and her heterozygous parents for a pathogenic *SPINK1* variant (rWGS_21).

Genetic counseling was offered to all diagnosed families, especially the family with exocrine pancreas insufficiency since heterozygous carriers of the mutation are symptomatic as well.

## 3. Discussion

We present the implementation of the first rapid Whole Genome Sequencing program for severely ill pediatric patients in Belgium. This study was designed as a proof of concept for the feasibility, the efficiency and utility of rWGS in Belgium.

### 3.1. Feasibility

Twenty-one probands were recruited within 21 months for the project, among which 16 were from the main study site, the CHR de Liège. Our recruitment pace was 0.76 patients per month at the main study site and one patient per month over the four recruitment sites. The Baby Bear project in California (USA) recruited 184 infants over eighteen months, corresponding to two patients per hospital per month [19]. Implementing rWGS with the TAT achieved in this study was challenging and demanded multiple adjustments to avoid delays (use of an easy-to-score checklist and direct pick-up of samples from the pediatric service between 8:00 and 8:30) and to prevent accumulation of work overtime for the personnel (implementation of flexible schedules for people involved in the wet lab and bioinformatics analysis). Although our recruitment pace may increase with the clinical utility being demonstrated, we recommend a semi-centered model for Belgium. In this model, one lab will be in charge for the wet lab. The other genetic laboratories may opt to outsource clinical interpretation as well or performing this last step in-house. A similar approach is applied by the Australian Genomics Health Alliance Acute Care [20].

### 3.2. Yield

Twelve definite diagnoses were made in the 21 patients (57.5%). This yield is among the highest reported in prospective rWGS studies, despite the small sample size [21]. Our current high yield may be the result of the inclusion of severely ill patients, combined with the administration of WGS as the first-tier test. Causal variants identified in this study were all single nucleotide variants located in the coding or splicing sequences. Although the benefits of WGS for CNV, SV, MT-SNVs and STRs analyses have been well documented [22], these approaches did not yield additional diagnoses in this study. A larger cohort will allow the evaluation of the added value of those approaches in Belgian patients.

In this cohort, one patient (4.76%) was affected by two distinct autosomal recessive disorders, both contributing to the phenotype. Double mendelian diagnoses are being documented in large studies of non-consanguineous patients with developmental disorders [23,24].

### 3.3. TAT

The TAT has become an important concern in genomic medicine. One of the major outcomes of the rWGS is the reduction of the TAT. However, most of the TATs reported in implementation studies similar to ours are often evaluated in a very controlled experimental design with fixed schedules for sample reception and processing [21,25], not addressing issues of human labor and laboratory work shifts, which may naturally generate delays due to evenings, weekends and holidays. To return a fast diagnosis, we applied on-demand sample processing, including during weekends, holidays, and evenings. We addressed human labor and laboratory work shifts as described in the feasibility subsection, above. Most samples were collected in the evening, because of the availability of parents.

Our TAT did not consider the delay caused by the unavailability of one parent or the transportation to the laboratory in the morning. We rather calculated the TAT based on the steps we could control, meaning from the sample reception to the report. We reduced the delay between sampling and reception in the laboratory by collecting samples the next morning from the three hospitals located in Liège, between 8 and 8:30. However, this approach may not be suited for big sample volumes. A study from Australia showed that for nationwide implementation, the distance between hospitals and the laboratory, and the means of transportation, may delay sample reception in the laboratory [20]. A nationwide implementation phase would need to address these issues upfront. Likewise, a nationwide implementation may also consider testing singletons or duos to avoid delays due to the unavailability of one or both parent(s).

We present the first implementation of rWGS in Belgium. We were able to return the diagnosis in about 40 h despite multiple manual steps. To the best of our current knowledge, this is the shortest delivery time for WGS in Europe. Several improvements are still possible, including the automation of data transfer between workflow steps, the use of FC S1 v1.5 200Cy in order to reduce sequencing run time from 25 h to 19 along with the sequencing cost.

### 3.4. Clinical Utility

The rWGS was very useful to this series of 21 patients. First, rWGS allowed correction of the diagnosis in multiple patients. The majority of clinical diagnoses were not confirmed by WGS. Unsuspected diagnoses were identified instead. This confirms previous observations that the clinical diagnosis is a poor predictor of the genetic findings in very young patients and supports gene-agnostic testing in NICU/PICU [5].

Second, the rWGS results informed the implementation of care, which included gene therapy, the use of an off-label drug in a trial, and disease-specific treatment. In the remainder, the multidisciplinary care was tailored to the diagnosis. The fact that all results were returned before the hospital discharge the integration of rWGS results in the care and disease surveillance. This represents the biggest advantage of fast-tract analysis as opposed to the standard of care.

Generally, multidisciplinary care may include speech therapy, physical therapy, behavior therapy, specialized education program, dietary care, medications for neurological manifestations and specific surveillance. Although multidisciplinary care may be initiated based on the clinical manifestation, before the diagnostic is known, multidisciplinary care is not generic. It varies depending on the diagnosis. In this study, the majority of patients did not have a multidisciplinary care plan in place yet at the time of diagnosis, probably due to their younger age. The diagnosis allowed a multidisciplinary care plan to be started.

### 3.5. Challenges/Limitations

The amount of overtime and the cost are the two main limitations to the broad implementation of rWGS in Belgium. In almost all patients, libraries were loaded onto the NovaSeq after office hours, between 8:00 p.m. and 10:00 p.m., and the bioinformatic analyses started 25 h later, meaning the next night, and continued through the morning. For this pilot project, the overtimes were mitigated by using a flexible shift schedules, and the cost was covered by a grant from the government of Wallonia in Belgium. The current reagent price, including TVA, is EUR 6124 for a trio. The semi-centered model will allow the pooling of up to six trios on a single S4 FC PE300 cycle, thus reducing the reagent costs. Ultimately, a specific reimbursement by the health system, as previously implemented for Non-Invasive Prenatal Testing (NIPT) [26], will offer equal access to the Belgian population given the undeniable benefit of rWGS.

## 4. Materials and Methods

### 4.1. Patients

Patients were recruited between February 2021 and October 2022. The main study site was the CHR de Liège, an affiliate hospital to the University hospitals of Liège, where 16 probands were recruited. Three other hospitals also contributed patients to the study: the Notre Dame des Bruyères with one patient, the CHC Mont-Légia with three patients, and the University Hospitals of Saint-Luc with one patient.

We used a recruitment checklist adapted from the Blue Shield checklist (checklist in the supplemental material). To be included, a patient had to (1) be admitted to NICU, PICU, or NEURO; (2) meet at least one inclusion criterion and have none of the exclusion criteria. (3) Babies born following sperm or egg donation, or who had been adopted, were excluded. In addition, (4) the patient’s clinical condition must permit drawing at least 1 mL of venous blood.

The neuropediatric unit is a branch of the pediatric service of the University Hospital of Liège (located in the CHR de Liège) where pediatric patients with neurologic pathologies or neurological phenotypes are admitted and treated. Those patients are coming from the pediatric emergency room, the pediatric intensive care unit or external referrals for neurologic assessment. This unit is composed of 16 beds and a certified neuropediatricians is always on duty.

### 4.2. Study Workflow

**Figure 3:**
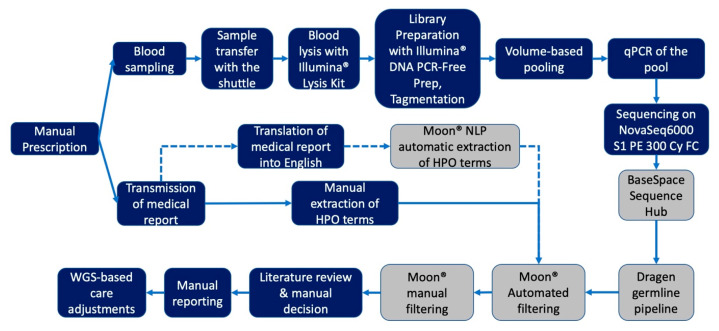
Workflow diagram of the pilot rWGS study. Legend: grey shapes represent steps performed using Cloud-based bioinformatic tools and platforms.

We combined an expedited test prescription approach with multiple cloud-based data analysis platforms into a single data workflow (Figure 3). This study workflow is described in detail in the Appendix A.

As opposed to the Australian model [20], where the mean sample transportation time was 1.6 days, the most distant points in Belgium are located within a radius of 2 h and half around our sequencing facility.

### 4.3. Library Preparation and Sequencing

Blood samples were lysed using the Illumina Lysis Kit (catalogue number 20042221) following the manufacturer’s protocol with the exceptions that we started with 40 µL of blood instead of 20 µL, as recommended, and we doubled the lysis solution’s volume. Lysis product was purified with Illumina purification beads (provided with the lysis kit) as recommended by the manufacturer and beads were resuspended in 30 µL of resuspension buffer provided in the lysis kit. DNA content in the lysis product was assayed with the Qubit fluorometer 4.0 and then normalized to the concentration of the least concentrated sample in the trio (see Supplementary Materials). Then, 30 µL of normalized lysis product was used for library preparation with Illumina DNA PCR-Free Prep, Tagmentation library preparation kit (Cat number 20041795) and IDT^®^ for Illumina^®^ DNA/RNA Unique Dual Indexes Set A, Tagmentation (96 Indexes, Cat number 20027213). Library preparation was performed manually in a diagnostic grade pre-PCR room using a Veriti^TM^ Thermal Cycler instrument and standard laboratory equipment. Libraries were pooled per volume, as recommended. From rWGS_3, we also applied the unique dual index correction factor as recommended by Illumina in order to improve the balance of the coverage across samples within the same run [27]. No intermediate Quality Control was performed. The volume to be loaded on the flow cell was determined by either Qubit fluorometer 4.0 (for rWGS_1) or qPCR of the pool (from rWGS_2). Between 1.27 to 1.87 nM of pooled libraries were loaded onto a Flow cell. After rWGS_1, S1 FC version 1.0 (Cat number 20012863) was discontinued by the manufacturer, and we shifted to S1 PE300cy FC v1.5 (Cat number 20028317). For the duo, (rWGS_6) SP v1.5 PE 300 cycles was used instead. Sequencing was performed on a NovaSeq6000 using a standard workflow with PhiX Control v3 (Cat number FC-110-3001). Despite variations in the percentage of reads passing filter, duplicate rates, and yield imbalance across samples, we achieved a mean coverage over the genome between 39 and 75×. The lowest coverage was achieved in rWGS_6 and rWGS_12, which were sequenced on a SP flow cell.

### 4.4. Analysis of Genomic Data

The standard duration of sequencing for an S1 PE300cycles is 25 h. Data transferred to the local server was used for backup (after demultiplexing) and the review of InterOp files using the Sequencing Analysis Viewer (SAV) Software v2.4.7 (Illumina, San Diego, CA, USA). A Base Space Sequencing Hub (BSSH) connection was established on the NovaSeq 600, allowing data to be directly streamed to the Cloud. BSSH is a bioinformatic environment that contains software and hardware tools such as bcl2fastq and the DRAGEN. Our data were hosted in the European instance of the BSSH, in Frankfurt. Upon the completion of the sequencing run and subsequent data transfer to BSSH, bcl2fastq was automatically launched for the generation of FASTQ files in BSSH. The output Fastq files were stored in BSSH and the research team was automatically notified by email upon the completion of the bcl2fastq. Next, DRAGEN Germline Pipeline 3.2.8 (DRAGEN Host Software Version 05.011.281.3.2.8 and Bio-IT Processor Version 0x04261818) was launched manually in BSSH for SNV and CNV detection. Dragen germline Pipeline 3.2.8 is highly optimized for speed and performs alignment and variant calling with high sensitivity and accuracy [28,29]. We aligned our patients’ data on the GRCh37 reference genome (3.1 Gb). In addition to SNV and CNV detection from nucleic DNA, this pipeline contains multiple additional but optional analysis modules such as structural variation detection, detection of Runs Of Homozygosity (ROH), and detection of repeat expansion analysis (short tandem repeats, STRs) using Expansion Hunter [6]. The output files consisted of vcf files, as well as cram files and related index files.

All molecular findings were validated with Sanger sequencing.

### 4.5. Phenotype Based Clinical Interpretation

The vcf files produced by DRAGEN were imported in Moon^®^ v3.3.2 (Invitae, San Francisco, CA, USA). This platform uses Artificial Intelligence and multiple knowledge sources to conduct phenotype-based filtering. First, Moon performs an autonomous analysis and provides a shortlist of variants best matching the phenotype (HPO terms) provided by the user along with genomic data. MOON uses a probabilistic model to rank the pathogenicity of variants retained during the autonomous filtering. This framework takes into account 15 in silico prediction tools, ClinVar records, expected inheritance pattern and inheritance pattern–based allele frequencies [12]. In addition, the user has the possibility of performing manual filtering of variants based on the depth, genotype quality, the frequency in gnomAD, the predicted effect, the inheritance, the zygosity and the region or gene of interest. Moon has been optimized for import and analysis of WGS data in short time. The following annotation sources were used in Moon: ClinVar: 21 November 2020, dbNSFP: 4.0, dbSNP: 151, dbscSNV: 1.1, Apollo: 26 November 2020, RefSeq: 37, gnomAD: 2.1.1, HPO: 12 February 2019, Moon KB: 9 March 2021, DGV: 1 March 2016, dbVar: 7 July 2019, Mitomap: 14 January 2019, Mitimpact: 2.9.1, Mastermind: 2 October 2020, InvitaeKB: 2 December 2020.

This pilot study focused on primary findings. Therefore, consent for analysis of incidental findings was not sought in the consent form

### 4.6. Outcome Measures for the Clinical Utility

The primary outcomes were the diagnostic yield and the TAT. For such a multisite recruitment, the most accurately recordable initial point is the time of sample reception by the laboratory which was thus considered the starting time. The final time point was the electronic signature of the analysis report. Those time points were collected from the local laboratory information management software.

Sanger sequencing was not a requirement before returning the results, since we knew, based on our local experience, that false positive SNVs were very unlikely with our coverage of 39× to 75×. However, given that our genetic service is accredited and that this rWGS is not accredited yet, our results had to be validated by an accredited test (sanger sequencing) to be included in the EHR. We performed Sanger sequencing and added a complementary paragraph to the report with the Sanger results. The time for the sanger sequencing was beyond out TAT.

Other outcomes included the molecular diagnostic yield and the clinical utility. The changes in clinical management were reported by referring physicians using a clinical utility survey form adapted from Scocchia et al. [30]. The level of satisfaction was not assessed during this pilot phase.

## 5. Conclusions

We have conducted the first implementation study of rapid Whole Genome Sequencing in Belgium. Twenty-one patients aged 2 days to 18 years were recruited. Our average TAT of 39.80 h (ranges, 37.05–43.7) is the fastest rWGS platform in Europe. We achieved a yield of 57.5% (12/21), one of the highest in Europe. rWGS triggered care adjustments in diagnosed patients, including clinical trials, condition-specific treatment, and multidisciplinary care. This study establishes the feasibility and demonstrates the utility of rWGS in Belgium.

## Figures and Tables

**Table 1:** Demographic details.

	rWGS_1	rWGS_2	rWGS_3	rWGS_4	rWGS_5	rWGS_6	rWGS_7	rWGS_8	rWGS_9	rWGS_10	rWGS_11	rWGS_12	rWGS_13	rWGS_14	rWGS_15	rWGS_16	rWGS_17	rWGS_18	rWGS_19	rWGS_20	rWGS_21
Age in year (days)	0.24 (86)	1.25 (457)	9.33 (3408)	0.03 (11)	0.05 (20)	18.31 (6688)	0.08 (28)	0.03 (11)	1.67 (609)	0.02 (9)	0.01 (2)	1.28 (467)	0.39 (142)	0.07 (25)	0.47 (170)	1.25 (457)	0.07 (24)	0.27 (100)	0.05 (18)	1.32 (481)	5.37 (1963)
Gender	F	M	F	M	M	M	F	F	F	F	M	F	M	F	M	F	M	F	F	M	M
Recruitment unit	PICU	NEURO	NEURO	NICU	NICU	PICU	NICU	NICU	NEURO	NICU	NICU	NEURO	NEURO	NICU	PICU	NEURO	NICU	PICU	NICU	PICU	PICU

Abbreviations: NICU, neonatal intensive care unit; PICU, pediatric intensive care unit.

**Table 2:** Molecular findings and HPO terms used in the clinical interpretation.

Patients	Pedigree	Phenotype	OMIM Diseases	Gene	Variant Coordinate (GRCh37)	HGVS c.	Protein Position	Zygocity	Inheritance	ACMG-AMP Classification
rWGS_1	Trio	Muscle hypotonia; Abnormality of brainstem morphology; Frontotemporal cerebral atrophy; Encephalopathy; Progressive encephalopathy; Apneic episodes in infancy; Strabismus; Nystagmus; Abnormal corpus callosum morphology; Enlarged sylvian cistern; Laryngomalacia; Abnormal eye movements	KLF7-related Developmental delay/intellectual disability with neuromuscular and psychiatric symptoms	*KLF7*	2:207,953,249	NM_003709.4: c.790G > A	p.Asp264As	Heterozygous	De novo	Pathogenic
rWGS_2	Trio	Seizures; Hypotonia, early; Hypotonia, generalized; Large fontanelles; Neurodevelopmental delay; Respiratory infections in early life; Recurrent respiratory infections; Dystonia; Increased serum lactate; Tented upper lips vermilions; Anteverted nares; High palate	Aromatic L-amino acid decarboxylase deficiency (AADCD)	*DDC*	7:50,566,899	NM_001082971.2: c.823G > A	p.Ala275Thr	Heterozygous	Paternaly inherited	Pathogenic
					7:5,054,432	NM_005859.5: c.1037A > G	p.Tyr346Cys	Heterozygous	Maternaly inherited	Likely Pathogenic
rWGS_3	Trio	Mixed demyelinating and axonal polyneuropathy; Progressive polyneuropathy; Progressive degeneration of movement; Difficulty in walking; Progressive muscle weakness; Feeding difficulties; Weight loss; Sensorimotor polyneuropathy affecting arms more than legs; Motor conduction block; Gastroesophageal reflux; Quadriparesis; Toe-walking	Unsolved							
rWGS_4	Trio	Generalized hypotonia; Generalized neonatal hypotonia; Neonatal hypotonia; Severe muscular hypotonia; Respiratory distress	Neurodevelopmental disorder with neonatal respiratory insufficiency, hypotonia, and feeding difficulties	*PURA*	5:139,494,456–139,494,458	NM_005859.4: c.697_699delTTC	p.Phe233del	Heterozygous	De novo	Pathogenic
rWGS_5	Trio	Chylothorax; Facial dysmorphism	Lymphedema, hereditary, III	*PIEZO1*	16:88,851,308	NM_001142864.4: c.64 + 1G > A		Heterozygous	De novo	Pathogenic
					16:88,792,770	NM_001142864.4: c.3890T > C	p.Leu1297Pro	Heterozygous	Paternaly inherited	VUS
					16:88,783,093	NM_001142864.4: c.6800C > T	p.Thr2267Met	Heterozygous	Maternaly inherited	VUS
rWGS_6	Duo	Brain calcification; Intellectual impairment; Thrombocytopenia; Leukopenia; Hepatic cirrhosis; Brain imaging abnormality; Neurodevelopmental delay; Retinitis pigmentosa; Speech delay	COACH syndrome 1	*TMEM67*	8:94,811,875	NM_153704.6: c.2130G > A	p.Met710Ile	Homozygous	Biparental	VUS
			Urbach-Wiethe disease	*ECM1*	1:150,480,710	NM_004425.4: c.25T > A	p.Leu9Met	Homozygous	Biparental	VUS
rWGS_7	Trio	Intrauterine growth retardation; Hydrocephaly; Aqueduct of sylvius stenosis; Severe intrauterine growth retardation; Leukopenia; Cholestasis; Thrombocytopenia; Neutropenia; Metabolic acidosis; Atrial septum defect	Unsolved							
rWGS_8	Trio	Severe muscular hypotonia; Lactic acidosis; Hyperammonemia, acute; Hyperammonemia; Neonatal asphyxia	Unsolved							
rWGS_9	Trio	Renal insufficiency; Failure to thrive; Hyperlaxity; Hemiparesis; Unilateral multifocal epileptiform discharges; Hemiclonic seizures; Seizure; Global developmental delay	Mitochondrial DNA depletion syndrome 4A (Alpers type)	*POLG*	15:89,864,238	NM_002693.3: c.2740A > C	p.Thr914Pro	Heterozygous	Maternaly inherited	Pathogenic
					15:89,870,432	NM_002693.2: c.1399G > A	p.Ala467Thr	Heterozygous	Paternaly inherited	Pathogenic
rWGS_10	Trio	Abnormality of the brainstem white matter; Increased urine alpha-ketoglutarate concentration; Leukodystrophy; Multifocal seizures; Generalized clonic seizure; Tonic-clonic convulsions; Generalized convulsive status epilepticus; Bilateral tonic-clonic seizure with generalised onset	Unsolved							
rWGS_11	Trio	Brain imaging abnormality; Increased troponin i level in blood; Abnormal metabolic brain imaging; EEG with burst suppression; Opacification of the corneal epithelium; 3-methylglutaric aciduria; Lactic acidosis; Leukoencephalopathy; Abnormality of the basal ganglia; Corneal opacity	Unsolved							
rWGS_12	Duo	High arched palate; Hypertelorism; Hypotonia; Facial dysmorphism; Abnormal interventricular septum morphology; Poor speech; Failure to thrive	Noonan syndrome 1	*PTPN11*	12:112,915,524	NM_002834.5: c.923A > G	p.Asn308Ser	Heterozygous	Absent in mother, father not available for testing	Pathogenic
rWGS_13	Trio	Hypernatremia; metabolic acidosis; generalized seizures, loss of consciousness	Unsolved							
rWGS_14	Trio	Cholestasis; Aortic valve stenosis; Conjugated hyperbilirubinemia; Jaundice; Episodic vomiting; Feeding difficulties in infancy; Meconium ileus; Elevated gamma-glutamyltransferase level; Prolonged neonatal jaundice; Nasogastric tube feeding in infancy	Alagille syndrome 1	*JAG1*	20:10,621,792	NM_000214.3: c.3016_3017delCC	p.Pro1006PhefsTer5	Heterozygous	De novo	Pathogenic
rWGS_15	Trio	Retinal dystrophy; Generalized hypotonia; Tracheomalacia; Sensorineural hearing impairment; Generalized neonatal hypotonia; Ptosis; Unilateral ptosis; Feeding difficulties; Absent or decreased deep tendon reflexes; Respiratory distress	Unsolved							
rWGS_16	Trio	Hypoplasia of the maxilla; Rhinitis; Central apnea; Hypotonia; Growth delay; Hypothermia; Night sweats; Dysautonomia; Failure to thrive; Cerebellar hemorrhage; Delayed gross motor development	Unsolved							
rWGS_17	Trio	Thick vermilion border; Plagiocephaly; Polyhydramnios; Seizure; Hypotonia; Prominent nose; Respiratory distress; Anteverted ears; Preauricular pit; Broad nose; Thick upper lip vermilion; Status epilepticus	Epileptic encephalopathy, early infantile, 14	*KCNT1*	9:138,656,902	NM_020822.3: c.1061T > A	p.Met354Lys	Heterozygous	De novo	Likely Pathogenic
rWGS_18	Trio	Dysphagia; Respiratory distress; Stridor; Facial paralysis; Psychomotor retardation; Retrognathia; Camptodactyly; Agenesis of cerebellar vermis HP	*TUBB3*-related spectrum syndrome	*TUBB3*	16:90,001,644	NM_006086.4: c.785G > A	p.Arg262His	Heterozygous	De novo	Pathogenic
rWGS_19	Trio	Hypoglycemia; Metabolic acidosis; Lactic acidosis;3-methylglutaconic aciduria;Noncompaction cardiomyopathy; Neonatal hypoglycemia; Hyperammonemia	3-hydroxy-3-methylglutaryl-Coa lyase deficiency	*HMGCL*	1:24,143,241	NM_000191.3: c.272T > A	p.Val91Asp	Homozygous		Likely Pathogenic
rWGS_20	Trio	Renal insufficiency; Increased intracranial pressure; Lactic acidosis; Acute rhabdomyolysis; Convulsive status epilepticus; Recurrent hypoglycemia; Cerebral edema	Unsolved							
rWGS_21	Trio	Conjunctivitis; Failure to thrive; Macrocytic anemia; Steatorrhea; Abnormality of the face; Short stature; Decreased body weight; low vitamine A and D; hypoalbuminemia	Infantile isolated exocrine pancreatic insufficiency, *SPINK1*-related	*SPINK1*	5:147,211,114	NM_001379610.1: c.27delC	p.Ser10ValfsTer6	Homozygous	Biparental	Pathogenic

## Data Availability

Genomic variants supporting the diagnoses in this publication are submitted to ClinVar and accession numbers will be provided after the review. Reasonable requests for additional anonymized data may be sent to the corresponding author and will be considered in case per case.

## Supplementary Materials

The supporting information can be downloaded at: https://www.mdpi.com/article/10.3390/ijms24044003/s1.

## Appendix A

The patient selection was made by the pediatrician and the test prescription was confirmed by a clinical geneticist. The final inclusion decision was made by the research team based on the presence of at least one inclusion criteria and the absence of any exclusion criteria from our BlueShield-based study checklist (Supplemental Materials). The informed consent from the parents was collected by a clinical geneticist. Once the consent was obtained, blood samples were collected from the trio (affected child and both parents) and transferred to the genetic laboratory for library preparation and sequencing. The Sequencing instrument was set up to transfer sequencing data to our BaseSpace Sequence Hub (BSSH) account, Frankfurt instance (Illumina Inc.). In addition, raw sequencing data were also simultaneously transferred to the hospital server for backup. Demultiplexing was automatically performed in BSSH once the sequencing run and data transfer were finished and data were fully transferred to the Cloud. Fastq biosamples were manually uploaded to DRAGEN Germline Pipeline 3.2.8 (Illumina Inc.) within BSSH. Resulting vcf files for SNVs and CNVs were manually exported from Dragen^®^ to Moon^®^ (Invitae Inc.) and HPO terms were fed to Moon for phenotype-based clinical interpretation. For the last 3 patients, we tested the new clinical natural language processing (CNLP) feature in Moon, which allows the automatic extraction of HPO terms from the medical file. VCF files for SNVs and CNVs were manually moved from BSSH to Moon. We complemented the interpretation with manual filtering based on inheritance hypothesis and population frequencies. Plausible candidate variants were retained for manual literature and database review. The final plausible variants were manually classified according to the ACMG-AMP. A manual report was written and returned to the clinician for reverse phenotyping and action when possible.
